# Large hospital outbreak caused by OXA-244-producing *Escherichia coli* sequence type 38, Poland, 2023

**DOI:** 10.2807/1560-7917.ES.2024.29.22.2300666

**Published:** 2024-05-30

**Authors:** Radosław Izdebski, Marta Biedrzycka, Paweł Urbanowicz, Dorota Żabicka, Teresa Błauciak, Dorota Lechowicz, Beata Gałecka-Ziółkowska, Marek Gniadkowski

**Affiliations:** 1 Department of Molecular Microbiology, National Medicines Institute, Warsaw, Poland; 2 National Reference Centre for Susceptibility Testing, Department of Epidemiology and Clinical Microbiology, National Medicines Institute, Warsaw, Poland; 3 Bacteriological and Serological Laboratory, Multispecialist Hospital, Nowa Sól, Poland; 4 Hospital Infection Control and Prevention Team, Multispecialist Hospital, Nowa Sól, Poland

**Keywords:** OXA-244, *Escherichia coli*, ST38, Poland

## Abstract

In February 2023, *Escherichia coli* sequence type (ST) 38 producing oxacillinase 244 (OXA-244-Ec ST38) was detected from three patients in a hospital in western Poland. Overall, OXA-244-Ec ST38 was detected from 38 colonised patients in 13 wards between February and June 2023. The outbreak was investigated on site by an infection control team, and the bacterial isolates were characterised microbiologically and by whole genome sequencing. We could not identify the primary source of the outbreak or reconstruct the transmission sequence. In some of the 13 affected wards or their groups linked by the patients’ movement, local outbreaks occurred. The tested outbreak isolates were resistant to β-lactams (penicillins, cephalosporins, aztreonam and ertapenem) and to trimethoprim-sulfamethoxazole. Consistently, apart from *bla*_OXA-244_, all isolates contained also the *bla*_CMY-2_ and *bla*_CTX-M-14_ genes, coding for an AmpC-like cephalosporinase and extended-spectrum β-lactamase, respectively, and genes conferring resistance to trimethoprim-sulfamethoxazole, *sul2* and *dfrA1*. Genomes of the isolates formed a tight cluster, not of the major recent European Cluster A but of the older Cluster B, with related isolates identified in Germany. This outbreak clearly demonstrates that OXA-244-Ec ST38 has a potential to cause hospital outbreaks which are difficult to detect, investigate and control.

**Key public health message**

**What did you want to address in this study and why?**

Carbapenems are a group of last resort antimicrobials to treat infections. Since 2017–18, carbapenem-resistant *Escherichia coli* sequence type 38 (OXA-244-Ec ST38) has been spreading in Europe, mainly in the community. Here we investigated a hospital OXA-244-Ec ST38 outbreak, which occurred in western Poland in 2023.

**What have we learnt from this study?**

To the best of our knowledge, this outbreak with 38 cases in 13 wards of a hospital is the largest OXA-244-Ec ST38 outbreak reported in Europe. We could not identify the primary source or trace the transmission of the outbreak or stop the outbreak. The isolates did not belong to a recent major European epidemic lineage of OXA-244-Ec ST38 but to an older and more diversified one and were related closer to some isolates from Germany.

**What are the implications of your findings for public health?**

Dissemination of OXA-244-Ec ST38, including hospital outbreaks caused by this *E. coli* strain, is difficult to investigate and contain. Spread of these bacteria is of concern for public health because of their broad resistance to antibiotics, including the last resort drugs in the treatment of hospital infections.

## Background

*Escherichia coli* is one of the most common causes of community- and hospital-acquired infections. Treatment of these infections has become difficult due to increasing antimicrobial resistance (AMR) [1], especially resistance to oxyimino-cephalosporins and fluoroquinolones, the former mediated mainly by extended-spectrum β-lactamases (ESBLs) [1,2]. Resistance to carbapenems, the traditionally last resort antimicrobials against multidrug-resistant (MDR) Gram-negative bacteria, has been rare in *E. coli,* so far. In the European Union/European Economic Area (EU/EEA), the percentage of carbapenem-resistant invasive nosocomial *E. coli* isolates was 1.5% in 2022, and in most of the countries it was below 1% (https://atlas.ecdc.europa.eu/).

Carbapenem resistance in Enterobacterales relies largely on carbapenem-hydrolysing β-lactamases, i.e. carbapenemases, in Europe mainly the *Klebsiella pneumoniae* carbapenemase (KPC), Verona integron-encoded metallo-β-lactamase (VIM), New Delhi metallo-β-lactamase (NDM) and oxacillinase-48 (OXA-48)-type enzymes [3]. Organisms with oxacillinase (OXA)-48-like β-lactamases prevail among carbapenemase-producing Enterobacterales (CPE) in many European countries, including Spain, France and Germany. These carbapenemases have been primarily associated with imports from Türkiye, Middle East or northern Africa [4]. Their expansion in European hospitals has been based on dissemination of plasmids with *bla*_OXA-48-like_ genes and clonal spread of multiple lineages of *K. pneumoniae* [4-6]. A specific phenomenon has been the diffusion of an *E. coli* sequence type (ST)38 clone with chromosomal *bla*_OXA-48_-type genes, since 2010, observed usually in outpatients [7]. More recently, the proliferation of *E. coli* ST38 with *bla*_OXA-244_, a single mutant of *bla*_OXA-48_, coding for a notably weak carbapenemase, has been of considerable interest [8].

Since 2013, OXA-244-producing *E. coli* (OXA-244-Ec) has been detected in Germany [9] and the United Kingdom (UK) [10], but from 2017–18 it has rapidly spread across Europe, especially in Germany, France, the Netherlands and Denmark [11]. By whole genome sequencing (WGS), a specific OXA-244-Ec ST38 sub-lineage, Cluster A, has been identified. This sub-lineage, notably homogeneous and co-expressing the cefotaximase (CTX)-M-27 ESBL was first identified in 2016. Another sub-lineage, Cluster B, detected in 2013, is less frequent, more diversified genetically and correlates with the CTX-M-14 enzyme [11-16].

Two major factors, which are overlooked, contribute to the spread of OXA-244-Ec ST38. Firstly, identification of the isolates is difficult due to low OXA-244 carbapenemase activity and their regular susceptibility to clinically relevant carbapenems, imipenem and meropenem [8,11]. Secondly, OXA-244-Ec ST38 has been spreading mainly in the community [11], with only one, to our knowledge, nosocomial outbreak reported in Norway [15].

Polish hospitals manage healthcare-associated infections based on the National Act on Preventing and Combatting Infections and Infectious Diseases in Humans (Journal of Laws of 2022, item 1657) and European Centre for Disease Prevention and Control (ECDC) definitions (https://www.ecdc.europa.eu). Hospitals must develop and implement procedures for infection prevention and control, use personal and collective protection means, assess the risk of infection, monitor alert MDR pathogens (e.g. CPE), conduct microbiological diagnostics and analyse the epidemiological situation for optimising antimicrobial therapy and prophylaxis. Hospitals are obliged to notify and report infections and outbreaks of alert organisms to the State Sanitary and Epidemiological Inspection.

## Outbreak detection

Between 27 and 28 February 2023, putative carbapenemase-producing *E. coli* were detected from three patients treated in a hospital in western Poland. Since the patients were in epidemiologically linked wards (cardiac surgery, cardiology and cardiological intensive care unit (ICU)), identification of these three similar *E. coli* isolates was a signal of a possible outbreak.

Here we report on the investigation of the second and to the best of our knowledge, largest hospital OXA-244-Ec ST38 outbreak in Europe, identified recently in Poland, also the fourth identification of OXA-244-Ec ST38 in the country [11].

## Methods

### Setting

The hospital (Hospital A) is a general secondary care hospital with ca 450 beds. Surveillance for MDR pathogens is performed on a regular basis in accordance with the national regulations. On admission, a rectal swab is taken from any patient with a history of hospitalisation within the previous year and from any patient moved between wards of higher risk of CPE acquisition. Furthermore, patients are tested periodically during hospitalisation, once a week or a month, depending on the ward.

### Outbreak investigation

The signal of a possible outbreak was followed directly by notification to the State Sanitary and Epidemiological Inspection and start of the investigation by the Hospital Infection Control and Prevention Team on 6 March 2023. It included formulating case definitions, identification of possible outbreak cases, review of medical records of the cases, drawing the graphical timeline of case identifications, development and verification of hypotheses on the transmission routes, identification of possible risk factors of acquisition and risk-exposed patients, review of medical care and hygienic procedures, environmental screening and typing of bacterial isolates.

### Case and outbreak definitions

A case was defined as a patient colonised or infected with OXA-244-Ec and hospitalised in Hospital A from February 2023 onwards. A colonised case was defined as a patient with OXA-244-Ec cultured and without any symptoms of infection. An infected case was defined as a patient with laboratory-confirmed OXA-244-Ec, identified as the aetiological agent of the clinical symptoms. Outbreak was defined as detection of at least two epidemiologically related cases. Contacts were defined as patients staying in the same hospital room as a case.

### Sampling of patients and environment

The first cases were detected within routine screening and the following ones by an enhanced protocol, according to which, each patient was screened on admission and then weekly during hospitalisation. Contacts were sampled after detection of each case, three times with an interval of 48 h.

Environmental samples were taken from hospital rooms and shared areas, like diagnostic or physiotherapy areas, depending on the ward. In hospital rooms, samples were taken after discharge of a case and cleaning of the room. Contact surfaces in the direct vicinity of a case (e.g. bed, mattress, cabinet) and items of general use (e.g. hand hygiene equipment) were swabbed.

### Microbiological investigations

A smear of a swab was plated onto ChromID CARBA SMART plate (bioMérieux, Marcy l‘Étoile, France) and MacConkey agar (bioMérieux) with 10 µg ertapenem discs (Oxoid, Basingstoke, UK). Carbapenemase phenotypic detection included the EDTA double-disc test for metallo-β-lactamases [17], phenylboronic acid combined disc test for class A carbapenemases [18] and 30 µg temocillin disk (Oxoid) for OXA-48 types [19]. Carbapenemases were tested also by the NG-Test CARBA-5 lateral flow immunoassay (NG Biotech, Guipry-Messac, France). Species identification and antimicrobial susceptibility testing were performed using VITEK 2 (bioMérieux). Suspected *E. coli* isolates collected between February and June 2023 were shipped to the NMI for further characterisation.

Susceptibility testing was performed by broth microdilution using Sensititre EUGNF, GNX3F and EUMDRXXF plates (Thermo Fisher Scientific, Waltham, the United States (US)), the ComASP Cefiderocol test (Liofilchem, Roseto degli Abruzzi, Italy) and in-house temocillin plates and interpreted according to EUCAST clinical breakpoints v.13.1. (http://eucast.org).

### Molecular and genomic analysis

At the NMI, *bla*_OXA-48_-type genes were confirmed by PCR [20], followed by digestion of the amplicons with the FspBI restrictase (Thermo Fisher Scientific), distinguishing *bla*_OXA-244_ (no digestion) from *bla*_OXA-48_ (digestion). The isolates were sequenced with Illumina MiSeq (Illumina, San Diego, US), and the first isolate (5550–23) was sequenced also by MinION (Oxford Nanopore Technologies, Oxford, UK). Libraries for Illumina and MinION were prepared using NEBNext Ultra II DNA Library Prep Kit (New England Biolabs, Ipswich, US) and Rapid Barcoding Kit (Oxford Nanopore Technologies), respectively. Raw sequence reads were trimmed and filtered with Cutadapt 3.11 (https://cutadapt.readthedocs.io/en/stable/). Illumina contigs were de novo assembled with SPAdes 3.15.4 [21], and the hybrid assembly with MinION reads was done with Unicycler v0.4.8 [22].

The seven-loci multilocus sequence typing (MLST) was performed with the mlst tool (https://github.com/tseemann/mlst) [23]. The single nucleotide polymorphism (SNP) analysis was done using BioNumerics 7.6.3 (Applied Maths, Sint-Martens-Latem, Belgium). The first isolate (5550–23) was used as a reference and sequenced by both the short- and long-read technologies. The phylogenetic analysis, including all OXA-244-Ec ST38 genomes available in GenBank, was performed with Parsnp v.1.2 [24]. The genomes were extracted with the mlst (https://github.com/tseemann/mlst) and AMRFinderPlus (https://github.com/ncbi/amr) tools from all 217,882 *E. coli* genomes downloaded from GenBank on 27 August 2023 by the National Center for Biotechnology Information (NCBI) genome-download script (https://github.com/kblin/ncbi-genome-download). A phylogenetic tree was visualised with iTOL (https://itol.embl.de). Resistance genes were detected with AMRFinderPlus with the sequence identity and coverage cut-offs of 100%. Plasmid replicon types were identified by PlasmidFinder v.2.1 [25], and resistance genes were assigned to plasmids with AMRFinderPlus.

## Results

### Descriptive epidemiological analysis

**Figure 1:**
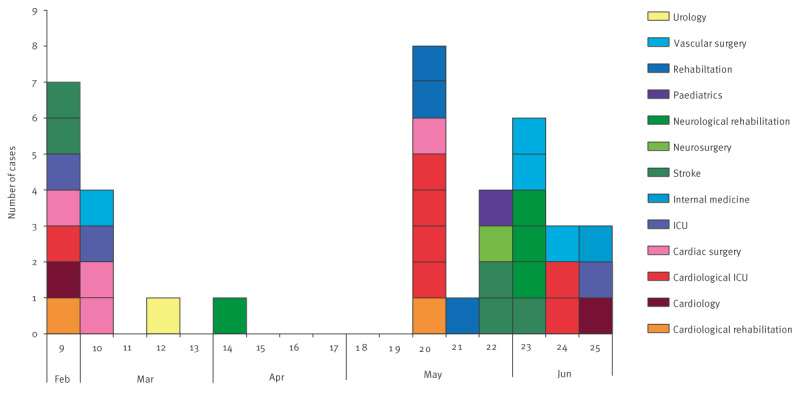
Confirmed cases with *Escherichia coli* sequence type (ST) 38 producing oxacillinase (OXA)-244 in a hospital outbreak, by week of sampling, Poland, February–June 2023 (n = 38)

In total, OXA-244-Ec ST38 was isolated from 38 colonised patients hospitalised in 13 wards of Hospital A between February and June 2023 (Table 1 and Figure 1). Of these cases, 27 were male and 11 were female. The age range of the patients was 2–97 years, with a mean of 67.2 and median of 70 years.

**Table 1:** Description of cases with oxacillinase (OXA)-244-producing *Escherichia coli* sequence type (ST) 38 in a hospital outbreak, Poland, February–June 2023 (n = 38)

Case ID	Isolate ID	Sampling month	Week	Hospital ward	Number of SNPs^a^
1	5550–23^b^	February	W9	Cardiac surgery^c^	0
2	5551–23	February	W9	Cardiology	0
3	5552–23	February	W9	Cardiological ICU	1
4	5553–23	March	W9	Cardiological rehabilitation	0
5	5554–23	March	W9	ICU	0
6	2080–23	March	W9	Stroke	0
7	2082–23	March	W9	Stroke	2
8	5555–23	March	W10	Vascular surgery	0
9	5556–23	March	W10	Cardiac surgery^c^	0
10	5557–23	March	W10	ICU	0
11	5558–23	March	W10	Cardiac surgery^c^	2
12	5559–23	March	W12	Urology	2
13	5560–23	April	W14	Neurological rehabilitation	9
14	5561–23	May	W20	Cardiological ICU	0
15	5562–23	May	W20	Cardiological ICU	0
16	5563–23	May	W20	Cardiological ICU	1
17	5564–23	May	W20	Rehabilitation	0
18	5565–23	May	W20	Cardiac surgery^c^	0
19	5566–23	May	W20	Rehabilitation	0
20	5567–23	May	W20	Cardiological ICU	1
21	5568–23	May	W20	Cardiological rehabilitation	0
22	5569–23	May	W21	Rehabilitation	1
23	5570–23	May	W22	Stroke	1
24	5571–23	May	W22	Stroke	2
25	5572–23	May	W22	Neurosurgery	0
26	5573–23	June	W22	Paediatrics	0
27	5574–23	June	W23	Vascular surgery	1
28	5575–23	June	W23	Vascular surgery	0
29	5576–23	June	W23	Neurological rehabilitation	1
30	5577–23	June	W23	Neurological rehabilitation	3
31	5578–23	June	W23	Neurological rehabilitation	0
32	5579–23	June	W23	Stroke	30
33	5580–23	June	W24	Vascular surgery	1
34	5581–23	June	W24	Cardiological ICU	1
35	5582–23	June	W24	Cardiological ICU	0
36	5583–23	June	W25	ICU	5
37	5584–23	June	W25	Cardiology	5
38	5585–23	June	W25	Internal medicine	0

ICU: intensive care unit; ID: identification code; SNP: single nucleotide polymorphism; W: week.

^a^ Number of SNPs compared with reference isolate 5550–23.

^b^ Reference isolate.

^c^ The cardiac surgery ward is an administratively independent unit located in the hospital building, collaborates strictly with the other wards and shares some personnel with those.

ICU: intensive care unit.

After detection of OXA-244-Ec from the index cases between 27 and 28 February, four new cases were detected by the end of the same week (W9) from three other wards (Figure 1). In W10, four new cases were detected, in W12 and W14, two cases were detected, and then a larger group of 25 cases were detected in W20–25. All cases were detected during hospitalisation, either within routine (in the initial phase of the outbreak) or enhanced (during outbreak investigation) testing.

As the cases were hospitalised in different wards, we could not identify the primary source of the outbreak, given that the first 11 cases were from seven wards. However, in some weeks, clusters of patients in the same ward were identified to be OXA-244-Ec ST38 carriers, like e.g. four patients in the cardiological ICU in W12, indicating local outbreaks. Moreover, some cases and hospital staff moved between certain wards involved in the outbreak, e.g. cardiology, cardiological ICU, cardiac surgery and cardiological rehabilitation, in all of which 16 colonised cases were hospitalised, including seven in the cardiological ICU. There were 10 colonised cases in stroke, neurosurgery and neurological rehabilitation units. It was not possible to identify the sequence of transmissions of the outbreak in the hospital.

The cases have not been specifically monitored after discharge. However, four of them were readmitted to the hospital at least 3   months later and were OXA-244-Ec-negative. The hospital personnel were not tested for OXA-244-Ec.

### Microbiological investigations

The *E. coli* isolates from the patients did not grow on the CPE selective medium but grew around ertapenem disks on MacConkey agar plates. The isolates were susceptible to imipenem and meropenem, but temocillin-resistant, suggesting the OXA-48-like carbapenemase production. The presence of OXA-48 was indicated by the NG-Test CARBA-5 lateral flow immunoassay.

The microbiology laboratory contacted the manufacturer of the chromogenic medium and upon request provided the company with two isolates for a basic WGS analysis. This identified the *bla*_OXA-244_ gene in the isolates, supporting the outbreak hypothesis and prompted the laboratory to contact the National Medicines Institute (NMI) in Warsaw for the analysis of all outbreak-associated isolates. The molecular (PCR and restriction digest) and genomic analyses in the NMI identified the OXA-244 enzyme in the isolates.

Environmental screening was performed in all wards with outbreak cases, and approximately 160 swabs were analysed altogether March–June 2023 but OXA-244-Ec was not cultured from any of the environmental samples.

### Clonality of the oxacillinase (OXA)-244-producing *Escherichia coli* sequence type 38 isolates

**Figure 2:**
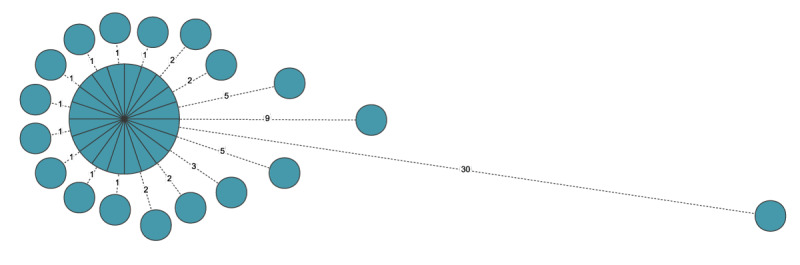
Single nucleotide polymorphism-based minimum spanning tree of *Escherichia coli* sequence type (ST) 38 isolates producing oxacillinase (OXA)-244 from a hospital outbreak, Poland, February–June 2023 (n = 38)

All 38 OXA-244-Ec ST38 isolates were whole genome sequenced. The SNP analysis revealed 68 polymorphic positions within ca 4.7 Mb (85%) of the reference genome. Individual genomes differed by 0 to 30 SNPs with the reference, and 20 isolates, including the reference, were indistinguishable from each other (Table 1 and Figure 2). The mean and median SNP values were 1.8 and 0, respectively.

SNP: single nucleotide polymorphism.

The minimum spanning tree was constructed using BioNumerics (Applied Maths).

Lengths of branches are related to numbers of SNPs in linked pairs of isolates (as shown on the branches).

### Phylogeny and international context of the outbreak isolates

**Figure 3:**
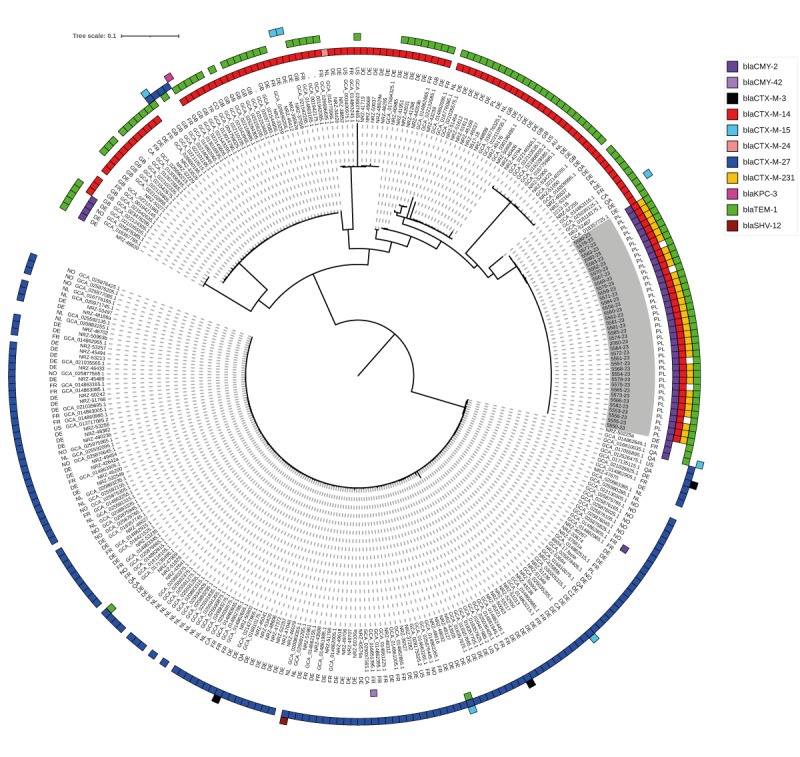
Single-nucleotide polymorphism-based phylogenetic tree of *Escherichia coli* sequence type (ST) 38 isolates producing oxacillinase (OXA)-244 from a hospital outbreak, Poland, February–June 2023 (n = 38), compared with other such isolates from GenBank (n = 164; as of 27 August 2023) and isolates from Poland (n = 3) and Germany (n = 100)^a^

AU: Australia; CA: Canada; CO: Colombia; CZ: Czechia; DE: Germany; FR: France; UK: United Kingdom; NL: the Netherlands; NO: Norway; PL: Poland; QA: Qatar; TR: Türkiye; US: the United States.

^a^ Isolates not submitted to GenBank.

We found 164 OXA-244-Ec ST38 genomes among the 217,882 *E. coli* genomes available in GenBank on 27 August 2023. The phylogenetic analysis of these 164 genomes, supplemented with 100 OXA-244-Ec ST38 genomes previously described in Germany [12] and the outbreak isolates, split the entire collection into two distinct major clusters. One of these was genetically homogeneous, correlated largely with the *bla*_CTX-M-27_ gene, and corresponded to Cluster A described in the recent ECDC rapid risk assessment (RRA) [11] (lower part of Figure 3). The second lineage was clonally more differentiated, usually contained the *bla*_CTX-M-14_ gene and correlated with Cluster B (upper part of Figure 3). The Polish outbreak isolates were located within Cluster B, forming a distinct branch with six related international isolates: three from Germany (36–39 SNPs distance to the Polish reference isolate), one from Canada (35 SNPs), one from Qatar (41 SNPs) and one from France (164 SNPs).

Numbers on the inner circle are the identification codes of the isolates from the hospital outbreak, GenBank assembly numbers and identification codes from a German study [12]. The large branch of genetically homogeneous isolates with the common presence of the *bla*_CTX-M-27_ gene, located in the lower part of the tree corresponds to Cluster A from a recent European Centre for Disease Prevention and Control (ECDC) rapid risk assessment (RRA) report [11], whereas the more diversified branch in the upper part corresponds to Cluster B. Our isolates are marked with grey. The tree was constructed using Parsnp and visualised with iTOL.

### Plasmids in *Escherichia coli* sequence type (ST) 38 isolates producing oxacillinase (OXA)-244

The combination of short- and long-read sequencing revealed a full set of plasmids in the reference OXA-244-Ec ST38 isolate (5550–23). There were three high molecular weight plasmids: an IncFII (pC15–1a [26]) type of 85,518bp, an IncFII, FIB and Col156 triple-replicon molecule of 134,075bp and a p0111-like plasmid of 96,127bp. The PlasmidFinder screening of the remaining genomes showed a minor variation in these, namely the lack of the pC15–1a-like FII replicon in five isolates. Only this IncFII-type plasmid had AMR genes.

### Resistomes and antimicrobial susceptibility

The resistome analysis of all 38 isolates showed a remarkable stability of the acquired AMR gene profiles in the isolates, with only slight variations from the presence or absence of few genes in the individual isolates and resulting in five profiles (Table 2). The most significant variation was demonstrated by the five isolates with the AMR profile II that lacked the pC15–1a-like IncFII-type plasmid, and, consistently, its *bla*_CTX-M-231_, *bla*_TEM-1_, *qnrS1*, *erm(B)* and *mph(A)* genes. Apart from those, the isolates were homogeneous in the β-lactamase genes content, including *bla*_OXA-244_, two ESBL genes *bla*_CTX-M-14_ and *bla*_CTX-M-231_ and the acquired AmpC-like cephalosporinase gene *bla*_CMY-2_. Based on long-read sequencing, the direct genetic context of the *bla*_OXA-244_ gene was determined in the reference isolate, being a truncated derivative of the Tn*1999.2* transposon inserted directly into the chromosome, identical to the variant ‘G’ reported in OXA-244-Ec ST38 from France [13].

**Table 2:** Resistomes of *Escherichia coli* sequence type (ST) 38 isolates producing oxacillinase (OXA)-244 in a hospital outbreak, Poland, February–June 2023 (n = 5)

Isolate ID	Acquired antimicrobial resistance genes						AMR profile	
	β-lactams	Aminoglycosides	Fluoroquinolones	Sulphonamides	Trimethoprim	Macrolides	Profile	n
5550-23	*bla*_CMY-2_ *, bla*_CTX-M-14_ *, bla*_CTX-M-231_ *, bla*_OXA-244_ *, bla*_TEM-1_	*aadA1, strA, strB, sat2*	*qnrS1*	*sul2*	*dfrA1*	*erm(B), mph(A)*	I	30
5553-23	*bla*_CMY-2_ *, bla*_CTX-M-14_ *, bla*_OXA-244_ *, bla*_TEM-1_	*aadA1, strA, strB, sat2*	ND	*sul2*	*dfrA1*	ND	II	5
5558-23	*bla*_CMY-2_ *, bla*_CTX-M-14_ *, bla*_CTX-M-231_ *, bla*_OXA-244_ *, bla*_TEM-1_	*aadA1, strA, strB, sat2*	*qnrS1*	ND	*dfrA1*	*erm(B), mph(A)*	III	11
5578-23	*bla*_CMY-2_ *, bla*_CTX-M-14_ *, bla*_CTX-M-231_ *, bla*_OXA-244_ *, bla*_TEM-1_	*aadA1, strA, strB, sat2*	ND	*sul2*	*dfrA1*	*erm(B), mph(A)*	IV	1
5579-23	*bla*_CMY-2_ *, bla*_CTX-M-14_ *, bla*_CTX-M-231_ *, bla*_OXA-244_ *, bla*_TEM-1_	*aadA1, aadA5, aac(3)-IId, strA, strB, sat2*	*qnrS1*	*sul1, sul2*	*dfrA1, dfrA17*	*erm(B), mph(A)*	V	1

ID: identification code; ND: not detected.

Resistance genes were identified with AMRFinderPlus with 100% coverage and sequence identity cut-offs.

Five isolates representing all identified AMR gene profiles were selected. The last column shows the number of isolates per antimicrobial resistance profile.

Antimicrobial susceptibility was tested for five isolates representing the five AMR gene profiles (Table 2). Like the general resistome homogeneity, the isolates were notably uniform in susceptibility patterns (Table 3): β-lactam resistance reflected the activity of the CTX-M-like, CMY-2 and OXA-244 enzymes, conferring resistance to penicillins, cephalosporins, aztreonam and ertapenem and susceptibility to imipenem, meropenem, cefiderocol, ceftazidime-avibactam, imipenem-relebactam and meropenem-vaborbactam. Of the other tested antimicrobials, the isolates were resistant to trimethoprim-sulfamethoxazole, but all were susceptible to aminoglycosides, fluoroquinolones, tigecycline, eravacycline, colistin, nitrofurantoin and fosfomycin.

**Table 3:** Antimicrobial susceptibility of *Escherichia coli* sequence type (ST) 38 isolates producing oxacillinase (OXA)-244 from a hospital outbreak, Poland, February–June 2023 (n = 5)

Isolate ID	Minimum inhibitory concentration (mg/L)																									
	AMP	AMC	TZP	TEM	CTX	CAZ	FEP	FDC	ATM	IMP	MEM	ERT	CZA	MVB	IPR	CIP	LEV	AMK	GEN	TOB	TIG	ERV	CST	NIT	SXT	FOS
5550-23	**> 16**	**> 32**	**> 64**	**> 64**	**> 32**	**> 16**	**> 16**	1	**32**	≤ 1	2	**> 2**	0.5	1	0.5	0.25	0.5	4	1	1	≤ 0.25	0.12	≤ 0.25	≤ 32	**> 8**	≤ 16
5553-23	**> 16**	**> 32**	**> 64**	**> 64**	**> 32**	**16**	**16**	0.25	**32**	≤ 1	0.5	**> 2**	≤ 0.25	0.5	0.25	0.25	≤ 0.25	≤ 2	≤ 0.5	≤ 0.5	≤ 0.25	0.12	≤ 0.25	≤ 32	**> 8**	≤ 16
5558-23	**> 16**	**> 32**	**> 64**	**> 64**	**> 32**	**> 16**	**> 16**	0.5	**32**	≤ 1	1	**> 2**	≤ 0.25	0.5	0.25	0.25	0.5	≤ 2	≤ 0.5	≤ 0.5	≤ 0.25	0.12	≤ 0.25	≤ 32	**> 8**	≤ 16
5578-23	**> 16**	**> 32**	**> 64**	**> 64**	**> 32**	**> 16**	**16**	0.5	**32**	≤ 1	0.5	**> 2**	≤ 0.25	0.5	0.25	0.25	0.5	4	≤ 0.5	≤ 0.5	≤ 0.25	0.12	≤ 0.25	≤ 32	**> 8**	≤ 16
5579-23	**> 16**	**> 32**	**> 64**	**> 64**	**> 32**	**16**	**16**	0.25	**32**	≤ 1	0.5	**> 2**	≤ 0.25	0.5	0.25	0.25	0.5	≤ 2	≤ 0.5	≤ 0.5	≤ 0.25	0.12	≤ 0.25	≤ 32	**> 8**	≤ 16

AMC: amoxicillin-clavulanic acid; AMK: amikacin; AMP: ampicillin; ATM: aztreonam; CAZ: ceftazidime; CIP: ciprofloxacin; CST: colistin; CTX: cefotaxime; CZA: ceftazidime-avibactam; ERT: ertapenem; ERV: eravacycline; FDC: cefiderocol; FEP: cefepime; FOS: fosfomycin; GEN: gentamicin; ID: identification code; IMP: imipenem; IPR: imipenem-relebactam; LEV: levofloxacin; MEM: meropenem; MVB: meropenem-vaborbactam; NIT: nitrofurantoin; SXT: trimethoprim-sulfamethoxazole; TEM: temocillin; TIG: tigecycline; TOB: tobramycin; TZP: piperacillin-tazobactam.

Resistant isolates are marked with bold. Testing was performed according to EUCAST recommendations (http://eucast.org).

## Outbreak control measures

A number of outbreak control measures were implemented in the affected hospital wards, including isolation of cases, contact screening with temporary isolation of the contacts, enhanced screening of each patient on admission and then weekly during hospitalisation, enhanced patient skin hygiene (with decontamination products and dedicated washing equipment), enhanced personnel hand hygiene (education, observation, monitoring of the skin disinfectant consumption), personnel contact precautions (disposable personal protection equipment), personnel dedication if possible, disposable or dedicated small medical equipment, increased environmental cleaning and decontamination (education, observation, monitoring of the consumption of cleaning and decontamination products, increased disinfection of contact surfaces), environmental screening for Enterobacterales and education of the personnel and informing patients and their visitors about detection of OXA-244-Ec and how to prevent its transmission. Educational activities were done by ward nurses using leaflets and oral consultations. Implementation of the individual control measures (e.g. personnel hand hygiene, hand hygiene equipment, patient isolation, contact precautions, handling of the hospital bedding) was evaluated in different wards several times, by observation of the staff by members of the infection control team and by control of the consumption of disinfectants, hand hygiene antiseptics and personal protection disposables (e.g. gloves). Apparently, the outbreak was not contained by the beginning of November 2023, given 14 new OXA-244-Ec patients were recorded from July to 5 November. However, clonality of these isolates has not been analysed by any molecular/genomic approach.

## Discussion

The expansion of OXA-244-Ec ST38 has been one of the most worrying phenomena in recent epidemiology of CPE in Europe, addressed in two consecutive ECDC RRAs in February 2020 and July 2021 [11] and in several reports from different countries [12-16]. This concern has arisen from the significance of *E. coli* as a major human pathogen which causes community-acquired infections [1] and from the role of carbapenems as the last line drugs in the treatment of severe MDR Gram-negative infections [3]. Even though the OXA-244-Ec ST38 isolates in our study were characterised by relatively limited AMR profiles, they were resistant to most of the commonly used β-lactams, including oxyimino-cephalosporins, and may thus contribute to further evolution towards extensive and higher-level AMR [8,11-16].

This pathogen, OXA-244-Ec ST38, is considered to circulate mainly in the community, which makes it especially difficult to track, investigate and control, augmented by difficulties with the laboratory detection of the organism, caused by low carbapenemase activity of OXA-244 and exemplified mainly by the lack of growth on CPE selective media [11]. Both factors may contribute to the likely unnoticed spread, which might be one of the key elements of its ecological and epidemiological success. Since 2017–18, OXA-244-Ec ST38 has been spreading in several European countries, including Germany, where it is now considered endemic [11-16,27]. Previous studies indicated that the spread of OXA-244-Ec ST38 was not associated with healthcare. Despite extensive efforts, sources and routes of transmission have remained unclear. Some hypotheses on food or travelling to endemic regions in north Africa and Middle East have been postulated, however, not supported by scientific evidence so far [11].

Until 2023, OXA-244-Ec ST38 had been detected from three infected or colonised patients in Poland, this information was included in the ECDC RRAs [11]. The cases in our study were not linked to the previous findings in Poland. This outbreak has increased the total number of notified OXA-244-Ec ST38 cases in the country.

To our knowledge, this report is the second hospital outbreak caused by OXA-244-Ec ST38, following the regional outbreak with 12 cases in three healthcare centres in Norway in 2020 [15]. In contrast to most European OXA-244-Ec ST38 isolates since 2018, including those from Norway, which belonged to the phylogenetic Cluster A, the Polish outbreak isolates belonged to Cluster B [11]. Cluster B has been occurring in Europe for a longer period than Cluster A and has been much more phylogenetically diverse with multiple sub-lineages observed in different countries, especially Germany and the UK, with isolates related to the Polish ones. Our study shows that Cluster B is able to spread in nosocomial settings.

Our and the Norwegian report demonstrate that, despite the relative rarity of *E. coli* nosocomial outbreaks in general, OXA-244-Ec ST38 has a significant ability to spread in hospital settings, which in part may be related to the diagnostic problems. Like in the community, the spread of OXA-244-Ec ST38 in both outbreaks must have been partially unnoticed, making the identification of sources and transmission networks impossible [15]. It should be underlined that hospital environments are favourable for AMR evolution; therefore, the nosocomial spread of OXA-244-Ec ST38 may accelerate segregation of its more resistant variants.

Despite multiple efforts, such as enhanced screening on admission, isolation of cases, enhanced contact precautions, and hand and hospital environment hygiene, most likely the outbreak was not yet under control at the time of submission. This so far failure in containment of the outbreak has indicated that the sampled and studied OXA-244-Ec ST38 isolates were not fully representative for the actual transmission network in the hospital, which has been the major limitation of our analysis. This might have resulted directly from the limited sensitivity of rectal swabbing in the detection of CPE, the aforementioned specific problems with the OXA-244 laboratory identification and/or temporary gaps in the monitoring and/or infection control procedures, including incidental, unnoticed problems with compliance to the enhanced control measures. The negative results of the environmental screening are difficult to interpret, however, at least in part these might be related to the lack of the enrichment step in the culturing procedure. The OXA-244-Ec ST38 outbreak constitutes an obvious threat for other medical institutions and the community on the regional and broader scale.

## Conclusion

The large-scale ongoing spread of OXA-244-Ec ST38 has been a significant threat for public health in Europe. So far, this has been observed mainly in the community, indicating the risk of the diffusion of carbapenem resistance outside nosocomial environments. However, our and the previous Norwegian reports evidence the potential of OXA-244-Ec ST38 to cause hospital outbreaks. Both the community and the nosocomial dissemination of OXA-244-Ec ST38 have been difficult to track and contain, and one of the reasons are diagnostic problems with the detection of the organism.
